# The First 13 Years of “Percorso Giacomo”: Patients’ Outcomes

**DOI:** 10.3390/children13030389

**Published:** 2026-03-11

**Authors:** Francesca Catapano, Giacomo Sperti, Maria Bisulli, Luigi Tommaso Corvaglia, Chiara Locatelli, Elvira Parravicini

**Affiliations:** 1 Department of Medical and Surgical Sciences (DIMEC), University of Bologna, 40138 Bologna, Italy; 2 Neonatal Intensive Care Unit, IRCCS Azienda Ospedaliero-Universitaria di Bologna (A.O.U.BO), 40138 Bologna, Italychiara.locatelli@aosp.bo.it (C.L.); 3 Department of Gynecology, IRCCS Azienda Ospedaliero-Universitaria di Bologna (A.O.U.BO), 40138 Bologna, Italy; 4 Department of Pediatrics, Columbia University Irving Medical Center, New York, NY 10032, USA; ep127@cumc.columbia.edu

**Keywords:** perinatal palliative care, life-limiting conditions, life-threatening conditions, PPC program

## Abstract

Objectives: To report the outcomes of a population of fetuses and neonates with life-limiting (LL) or life-threatening (LT) diagnoses leading to adverse prognoses cared for by a service of perinatal palliative care (PPC), the Percorso Giacomo (PG). Study design: This is a single center retrospective cohort study of all fetuses and neonates prenatally or postnatally diagnosed with LL or LT conditions whose families opted to continue the pregnancy at IRCCS Policlinico di Sant’Orsola in Bologna, Italy, from 2013 to 2025. Results: There were 83 fetuses and/or neonates including 64 diagnosed prenatally and 19 postnatally with annual significant increments in number. All families encountered the PG team. Overall, the cohort demonstrated a very high cumulative rate of comfort care plan (90%) with high rate of redirection of goals of care from intensive to palliative. Conclusions: PG showed a significant growth over 13 years suggesting the strong need of a service of PPC. The continuity of care provided by PG facilitated parental decision-making process towards redirection of goals of care. The outcomes observed provided valuable insights related to the wide range of prognoses for each diagnosis that will enable more informed counseling in the future.

## 1. Introduction

Congenital anomalies are present in 2–3% of births and are a leading cause of morbidity and mortality [1]. Due to advancements in biotechnology, many of these conditions can be diagnosed prenatally. For families who choose to carry the pregnancy to term, it is crucial to develop a personalized care plan for the newborn. Moreover, newborns at the cusp of viability and those affected by serious conditions who fail intensive care may require redirection of goals of care to palliative care [2]. When the diagnosis is certain, a palliative care approach focused on the infant’s comfort is not only appropriate but essential [3,4,5,6,7].

The number of infants with life-limiting condition (LLC) and life-threatening condition (LTC) is significant and scientific evidence supporting the benefits of a perinatal palliative care (PPC) approach has been recognized by leading perinatal organizations such as the American College of Obstetrics and Gynecology (ACOG) [5], the American Academy of Pediatrics (AAP) [8] and the Italian Society of Neonatology (SIN) [9]. However, PPC remains difficult to access and is offered by only a very limited number of centers across Italy [10].

The “Percorso Giacomo” (PG) is a PPC service at the Istituto di Ricovero e Cura a Carattere Scientifico (IRCCS) Policlinico di Sant’Orsola in Bologna, Italy. The IRCCS is an academic institution that offers a busy service of maternal fetal medicine (MFM), a level III neonatal intensive care unit (NICU) and a large range of pediatric services. PG was established in 2013 with the goal of offering a safe, comforting, and compassionate environment for infants diagnosed with LLC and LTC, either prenatally or postnatally. The program is dedicated to assessing and ensuring each infant’s comfort, prioritizing their well-being at every stage of care and supporting families [11].

PPC has seen a remarkable expansion in terms of theoretical interest; however, the impact of a PPC dedicated service on the outcomes of pregnancies diagnosed with fetal LLC or LTC, and of infants postnatally diagnosed with LLC or LTC, is rarely reported.

This study aims to present the PG experience over the past 13 years.

Specifically, we hypothesize that more families referred to PG team would opt for a neonatal plan of comfort care or redirection of goals of care for their infants diagnosed with LLC and LTC when compared to what had been reported in the literature. We also hope this research will bring a better understanding of the wide range of prognoses for any given LL or LT diagnosis, to improve families’ counseling in the future.

## 2. Methods

This is a retrospective, descriptive study of a consecutive case series of pregnancies and infants born or transferred to the IRCCS, and followed by the PG team, as they had a prenatal or postnatal diagnosis of LLC or LTC from 1 January 2013 to 31 December 2025.

Maternal electronic medical records were reviewed to collect demographic data, fetal diagnosis and pregnancy outcomes. Neonates’ records were also reviewed, and the following data were collected: diagnosis, timing of diagnosis (prenatal vs. postnatal), sex, gestational age, postnatal care strategies, and age at death, when relevant. Data were accessible only to authorized personnel and were coded and entered into an anonymized Excel database. All data were analyzed using descriptive statistics.

Inclusion criteria:

Pregnancies with a fetal diagnosis of LLC and LTC in which parents elected to continue the pregnancy. Infants admitted to the NICU with a diagnosis of LLC and LTC and referred by the NICU physicians as candidates for redirection of goals of care.

Exclusion criteria:

Pregnancies with a fetal diagnosis of LLC and LTC in which parents elected to terminate the pregnancy. Infants admitted to the NICU with a diagnosis of LLC and LTC who were not candidates for redirection of goals of care.

### 2.1. PG Team

The PG at IRCCS is composed of a multidisciplinary team led by a neonatologist, who serves as medical director, a nurse coordinator, an obstetrician and a psychologist.

The PG team works closely with the MFM team, with the NICU team, and with consultants from other services, such as cardiology, surgery, neurology, etc., depending on the specific fetal or neonatal diagnosis.

The PG team meets with all families who have received a prenatal diagnosis of LLC or LTC and elect to continue the pregnancy. Moreover, the team receives referrals of NICU patients diagnosed with an LLC or a LTC and potential adverse outcome and offers palliative consults as an extra layer of care in addition to intensive medical treatment or facilitates redirection of goals from intensive to palliative care.

### 2.2. Comfort/Palliative Care

When an LLC or LTC is suspected during pregnancy, and the family elects to continue the pregnancy, the obstetrician refers the case to the PG team for a consultation [12]. During the initial consultation the suspected diagnosis and the corresponding wide range of prognoses are discussed. The team probes the family preferences and offers options for a personalized care plan that includes comfort/palliative care (CC) or resuscitation and intensive care (IC) if appropriate and requested by the family. If the LL diagnosis is confirmed postnatally, a shared comfort-focused plan is created to support the infant and family bonding, in the respect of the natural length of life.

The PG care approach prioritizes meeting neonates’ basic needs such as bonding, warmth, feeding and alleviating discomfort and pain [13,14]. PG guidelines, as previously described, align with these principles [11]. Each neonate’s care plan is personalized based on medical condition, gestational age, prognosis, and family preferences. All plans are documented in the medical record for team-wide access. The PG team tentatively attends every delivery of these patients, regardless of care decisions.

In case of intrauterine fetal demise (IUFD) or stillbirth the PG team is committed to supporting and caring for families.

### 2.3. Redirection of Goals of Care

When infants admitted to the NICU with life-threatening conditions fail medical interventions and/or the burden of care outweighs the benefit, forgoing such treatments may be ethically and clinically appropriate. The PG team facilitates the conversation with the NICU team and the family for a potential transition from IC to CC. This transition may happen in steps including a blend of both approaches, with ongoing reassessment of medical interventions. PG team also facilitates conversations in the NICU when families would like to transition the care of their infant from CC to IC.

## 3. Results

During the study, the PG team followed 83 patients from 81 families, including 2 twin pregnancies. Most referrals (64) occurred after a prenatal diagnosis of an LLC or LTC, while 19 were made postnatally. Overall, 53% of the newborns were male.

Table 1, Table 2 and Table 3 show the study population. Table 1 includes 19 pregnancies with fetal or intrapartum death. In all these cases parents had elected for a comfort-focused approach.

Table 2 includes 45 pregnancies with live births. At the prenatal counseling, 56% (25/45) of the families elected a CC plan; however, after the postnatal evaluation and confirmation of the diagnosis, the rate of CC plan increased to 82% (37/45).

In eight cases there was a change in plan of care from CC to IC. In four cases the postnatal evaluation demonstrated a clinical condition less severe than expected, including the diagnoses of Myhre syndrome, Tetralogy of Fallot + Truncus Arteriosus, Jeune syndrome, and bilateral renal dysplasia with normal lung function. All these infants are still alive at the time of the submission. In the other four cases, the parents opted for intensive care after delivery, despite confirmation of the diagnosis and prognosis. One infant diagnosed with Trisomy 18 lived for nine years and eventually died from respiratory failure. Another infant with Trisomy 18 complicated by myelomeningocele, hydrocephalus and HLHS failed resuscitation and died within a few hours. One infant was prenatally diagnosed with Harlequin ichthyosis. At the time of this submission she is 3 years old, needs complex daily dressing of the entire body for her severe skin condition, and has fair neurocognitive development. The fourth infant was diagnosed with Transposition of the Great Vessels and severe brain anomalies complicated by hydrocephalus. At the 6-month life follow-up the infant was still alive, however vent-dependent and with severely compromised neurological status.

Table 3 includes the postnatal referrals of 19 infants admitted to the NICU for IC whose plan of care was subsequently changed to CC because of postnatal diagnosis of LLC or LTC and failure of intensive care approach.

Out of the 64 infants prenatally or postnatally diagnosed with an LLC or LTC, only 8 are still alive and 6 have been managed with IC, as described above. Two patients were offered CC. At the time of this submission, one is 6 years old with a severe brain injury after perinatal HIE. She is quadriplegic, spontaneously breathing, fed by gastrostomy and followed at home by the pediatric hospice service. The other is 1 month old at the time of submission, has a diagnosis of hydranencephaly by MRI and has a flat EEG. She is spontaneously breathing, fed via a nasogastric tube and has been transitioned to an inpatient hospice.

All the NICU cases referred to the PG team (n = 19) underwent a redirection of goals from IC to CC. Among live-born infants (groups 2 and 3), the rate of comfort care was 89%. Overall, the entire cohort demonstrated a very high cumulative rate of comfort care plans (90%).

Most infants treated with CC directly from birth lived for hours; the longest survival time was 6 months.

**Figure 1:**
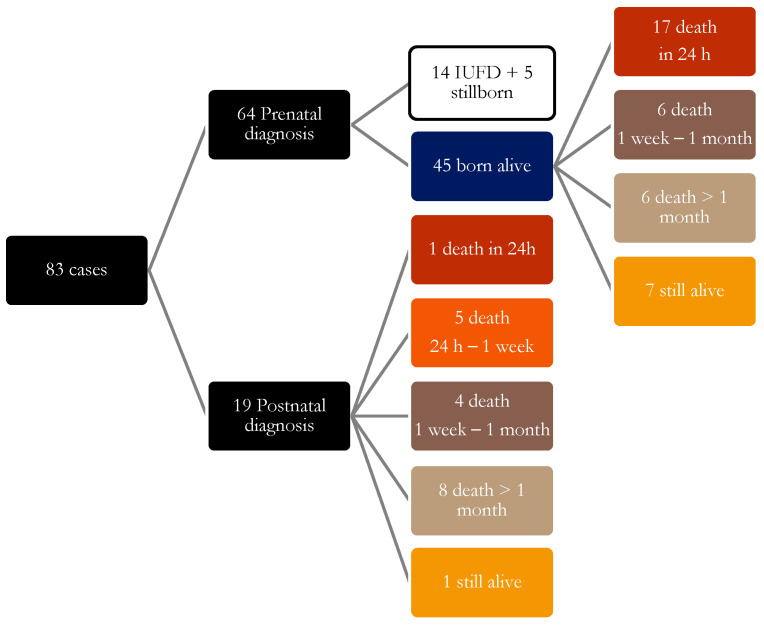
Patient outcomes following referral to the PG team.

Figure 1 summarizes outcomes and age at death or length of survival.

**Figure 2:**
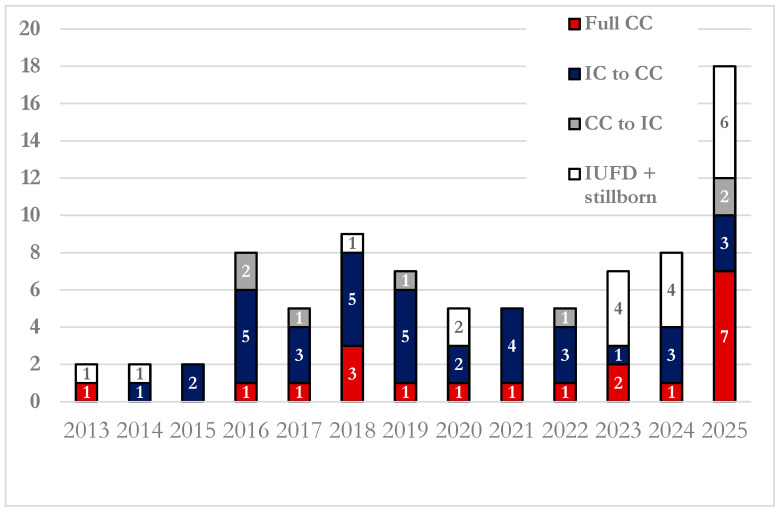
Annual distribution of cases followed by the PG team, stratified by plan of care.

The data were analyzed to assess the trend of cases over the years. Figure 2 shows the number of cases managed by the PG team annually from 2013 to 2025, highlighting the medical plan and the changes in number by year. Of note the cases increased constantly over time with only a temporary decline in the years 2020–2022, most likely due to the COVID-19 pandemic.

## 4. Discussion

To our knowledge this is the first report of the outcomes of a large cohort of fetuses and infants referred and managed by a structured perinatal palliative service in an academic institution in Italy over more than a decade. This single-center retrospective cohort study highlights the impact of a dedicated PPC team in the care of patients with fetal or neonatal diagnosis of LLC or LTC. This study also provides an overview of prenatal, perinatal and postnatal outcomes of fetuses and infants with LLC and LTC. Unique to this population is that all families who received a fetal diagnosis of potential LLC and elected to continue the pregnancy received counseling and discussed the infant’s postnatal care plan with the PG team. This consult rate sets this cohort apart from others published in the literature that report encounter rate and continuity of care with a PPC team of approximately 50% [15,16].

The outcome data over a 13-year period demonstrated a predominant use of comfort-focused care, with comfort care plans implemented in approximately 90% of cases and redirection from intensive to palliative care occurring in over half of patients. The comfort care rate in this population exceeds that reported in the existing literature [17,18,19,20]. We believe that this high rate of palliative care may be explained by the continuity of care assured by a dedicated team that operates in the hospital settings, not present in other centers [15,16]. In the experience of PG, engaging in a journey with families early in pregnancy was crucial to follow these families over time, supporting them through the evolution of the fetal diagnosis as well as the development of the parents’ wishes regarding how they wanted to care for their child. This extended time also helps foster a relationship of trust between the parents and the healthcare team [20]. The creation of a therapeutic bond with families is essential, and research consistently shows that early initiation of palliative care can substantially improve parents’ experience [21,22]. Unlike other programs [23,24], our team does not adhere to a predetermined number of meetings, adopting instead a flexible, family-centered approach based on the specific needs of each family. Toward the end of the pregnancy, a shared care plan is developed. This document outlines the clinical aspects of care including labor and delivery plan for the mother and the postnatal plan of care for the newborn. Moreover the document indicates parental preferences for caring for their baby, including memory-making, religious rituals, and other meaningful interventions. Very often parents have a quite short time with their baby, thus the multidisciplinary team of PG plays an essential role in directing goals of care and offering guidance and support. In this way the time parents have with their baby may be brief but deeply meaningful [25].

Additionally, 22% of these pregnancies resulted in IUFD and 8% in stillbirth. Thorough discussions following a fetal LLC or LTC diagnosis are essential, addressing the risk of IUFD and stillbirth in the settings of severe anomalies and unmonitored labor. It is essential to ensure that families are given adequate time and knowledge to fully understand all available options before making a decision. Evidence has shown that when families are offered the option of continuing the pregnancy within the framework of a structured perinatal palliative care plan, the majority choose to proceed with the pregnancy [26].

When a family followed by the PG team experiences either IUFD or stillbirth, the PG team still cares and supports the family, while the team is not involved in pregnancies that end in termination, in contrast to some reports in the literature [16]. As underscored by the ACOG [27], a clear distinction exists between termination of pregnancy and PPC pathway. However, families who elect termination of pregnancy should be equally supported in their grieving experience, clinical needs, follow-up for any future pregnancies and risk of recurrence, but managed by specific and structured service already present (or already existing) in the institution with other professionals with different and specific competencies [5,27].

The PG team was involved in conversations for redirection of goals of care in a number of NICU cases when the intensive treatment offered no real benefit to the infant, while imposing a high burden. This rate of redirection of care—higher than that reported in the literature [28,29]—is likely attributable to the presence of a dedicated palliative care team within the hospital.

As clearly represented in Figure 2, the number of cases requiring redirection of care remains consistently high each year. This result highlights the complexity of these clinical situations and, above all, the prognostic uncertainty that characterizes them. However, the same prognostic uncertainty has been widely recognized in the literature as a major barrier to the integration of palliative care [24,30]. Palliative care should not be delayed in the face of diagnostic or prognostic uncertainty; rather, it can—and should—be provided alongside intensive treatment when appropriate. In fact, early perinatal palliative care should become the standard of care not only for conditions with high mortality rates, but also for those involving prolonged hospitalizations, chronic clinical complexity, and the need for ongoing and difficult medical decision-making [15].

In our population, most neonates including those with prenatal diagnosis or postnatally referred with LLC or LTC (66%) died within the first weeks of life, with 43% of those deaths occurring within the first 24 h. This finding aligns with previously reported data [23]. Survival ranges widely, from only a few hours of life to several years. This variability depends on the clinical approach adopted—for instance, IC may prolong life expectancy—and, most importantly, on the specific prognosis within the same condition. For instance, infants affected by Trisomy 18 without cardiac or other major anomalies may have a significantly longer survival than those with associated organ dysfunction [31]. Therefore, careful evaluation is required, and the palliative care approach should be individualized in the interest of the proper care for each child.

In our population, some newborns initially received palliative care but were later transitioned to an intensive care approach. As shown in Figure 2, this is a relatively rare event, associated with two main factors: either a parental decision after they met their infant at birth or a postnatal diagnosis/prognosis that appeared less severe than expected. It remains essential to reassess the newborn’s clinical condition to ensure that all decisions are made in the best interest of the child. While advances in prenatal diagnostic techniques have greatly improved accuracy, as shown in our data with 95% of prenatal diagnoses confirmed postnatally, discrepancies can still occur [32].

The constant growth of referred patients over time, with only a brief decline at the time of SARS-CoV-2 pandemic, is likely due to the establishment of our center as a referral hub for perinatal palliative care, supported by a multidisciplinary team with specialized training. Moreover, in our academic institution there is strong presence of maternal fetal medicine and professionals of other specialties, such as pediatric surgery and cardiology, and others, concentrating a large number of high-risk pregnancies. Consequently, the increasing number of cases—combined with a more standardized and systematic implementation of palliative care as a valid scientific option—supports the expectation that this number will continue to grow, as evidenced by the trend observed over the past years.

Our multidisciplinary model, encompassing both clinical and supportive care, appears to closely reflect the eight domains of palliative care [33]. Greater awareness and integration of perinatal palliative care among healthcare professionals remain essential, so that it may be consistently offered as a valid option —alongside intensive care—to all families electing to continue a pregnancy complicated with an LLC or LTC.

This study has a few limitations. First, it reflects the experience of a single center, which may limit the generalizability of the findings. Although the cohort is large when compared with other Italian and European experiences, it remains smaller than cohorts reported from the United States [34] and other regions worldwide.

## 5. Conclusions

Over the past 13 years the PG program demonstrated a significant growth, underscoring the essential need for structured PPC services. The very high rate of CC in our population suggests that the continuity of care provided by the PG team along the perinatal journey facilitated the decision-making process, particularly with regard to the redirection of goals of care from intensive to a palliative approach. Lastly, the outcomes observed yielded valuable insights into the knowledge of the wide spectrum of prognoses associated with each individual diagnosis, thereby providing important data to ameliorate informed counseling for families facing LL or LT conditions for their infants.

## Figures and Tables

**Table 1:** Study population: Pregnancies with fetal or intrapartum death.

N° Patients	Sex	Prenatal Diagnosis	Outcome	Prenatal Plan of Care	Year of Birth
1	F	Cystic hygroma, multiple anomalies	IUFD	CC	2013
2	M	Trisomy 21, anasarca	IUFD	CC	2014
3	M	ALG3-congenital disorder of glycosylation	IUFD	CC	2018
4	F	Trisomy 21, cystic hygroma	IUFD	CC	2020
5	M	Trisomy 18, HLHS (Twin A)	IUFD	CC	2023
6	M	Multiple anomalies	IUFD	CC	2023
7	M	Severe IUGR	IUFD	CC	2024
8	F	Trisomy 18	IUFD	CC	2024
9	M	Trisomy 13, cleft palate, CHD	IUFD	CC	2024
10	M	IUGR, multiple anomalies	IUFD	CC	2025
11	F	Severe IUGR (twin A) in twin-to-twin transfusion syndrome	IUFD	CC	2025
12	F	HLHS (twin B) in twin-to-twin transfusion	IUFD	CC	2025
13	F	Monosomy X, severe IUGR	IUFD	CC	2025
14	F	Trisomy 18, Truncus Arteriosus, VSD, myelomeningocele	IUFD	CC	2025
15	M	TGV, right aortic arch, mitral atresia, enlarged bladder, PUV, anhydramnios, cerebellar vermis hypoplasia	stillborn	CC	2020
16	F	Trisomy 18, left diaphragmatic hernia	stillborn	CC	2024
17	M	Trisomy 13, omphalocele	stillborn	CC	2023
18	M	Tricuspid dysplasia, aortic hypoplasia, biventricular severely depressed function and failure	stillborn	CC	2023
19	M	Trisomy 13, aortic arch hypoplasia, VSD, hypoplasia of corpus callosum, cerebellar vermis	stillborn	CC	2025

IUFD: intrauterine fetal demise; CC: comfort/palliative care; HLHS: hypoplastic left heart syndrome; IUGR: intra-uterine growth restriction; CHD: Congenital Heart Disease; VSD: Ventricular Septal Defect; TGV: Transposition of the Great Vessels; PUV: Posterior Urethral Valves.

**Table 2:** Study population: Pregnancies with live births.

N° Patients	Sex	Prenatal Diagnosis	Outcome	Prenatal Plan of Care	Year of Birth
1	M	Anencephaly	19 h	CC	2013
2	M	Trisomy 21, severe hydrops	10 days	IC to CC	2015
3	F	Trisomy 18	31 h	IC to CC	2015
4	F	Anencephaly	1 h	CC	2016
5	M	Multiple anomalies	7 days	IC to CC	2016
6	M	Metabolic disease	7 days	IC to CC	2016
7	M	Jeune syndrome	still alive	CC to IC	2016
8	F	Trisomy 18	9 years	CC to IC	2016
9	M	Severe hydrops non isoimmune	7 days	IC to CC	2016
10	F	Spinal muscular atrophy, congenital fractures	2 months	IC to CC	2016
11	F	Left diaphragmatic hernia, HLHS	19 days	IC to CC	2017
12	F	Trisomy 18, myelomeningocele, hydrocephalous, HLHS	26 h	CC to IC	2017
13	F	Severe IUGR, prematurity (29 weeks GA), postnatal diagnosis of Myhre Syndrome	still alive	CC to IC	2017
14	F	Trisomy 18, myelomeningocele, hydrocephalus, VSD	6 months	CC	2018
15	F	Spinal muscular atrophy, congenital fractures (SMABF-1gene)	12 days	IC to CC	2018
16	M	Right diaphragmatic hernia	2 days	IC to CC	2018
17	F	Anencephaly	1 h	CC	2018
18	F	IUGR, skeletal anomalies (bilateral club feet, short long bones)	1 month	IC to CC	2018
19	F	Trisomy 18, DORV, PA, VSD	34 days	IC to CC	2019
20	F	Anencephaly	2 h	CC	2019
21	M	Bilateral renal dysplasia	1 h	IC to CC	2019
22	F	Truncus, TOF	still alive	CC to IC	2019
23	F	Trisomy 13, prematurity (25 weeks GA), hydrops	4 days	IC to CC	2019
24	M	ARPKD	1 h	CC	2020
25	M	ARPKD	19 h	IC to CC	2020
26	M	Trisomy 18, VSD, ASD	49 days	IC to CC	2021
27	M	Severe IUGR, prematurity (33 weeks GA) meconium ileum, cardiomegaly, tricuspid insufficiency, multiorgan failure	14 days	IC to CC	2021
28	M	Anencephaly (Twin B)	3 h	CC	2021
29	F	ARPKD	17 h	IC to CC	2021
30	M	Large-arteriovenous malformation of the brain with cardiac failure	1 h	CC	2022
31	F	Trisomy 18, tricuspid atresia	4 months	IC to CC	2022
32	F	Harlequin ichthyosis	still alive	CC to IC	2022
33	F	ARPKD	3 h	IC to CC	2022
34	F	ALG3-congenital disorder of glycosylation	3 h	CC	2023
35	M	Pfeiffer Syndrome	22 days	IC to CC	2023
36	F	Cerebral teratoma	3 h	CC	2024
37	F	Anencephaly	1 h	CC	2025
38	M	Anencephaly	30 h	CC	2025
39	F	Trisomy 18, HLHS	51 h	CC	2025
40	M	LUTO with normal lung function	still alive	CC to IC	2025
41	M	PSVT, cardiac failure and anasarca	1 h	CC	2025
42	F	TGV, severe hydrocephaly	still alive	CC to IC	2025
43	M	Bilateral renal agenesis	1 h	CC	2025
44	M	LUTO, anhydramnios	1 h	CC	2025
45	F	Hydranencephaly	still alive	CC	2025

CC: comfort/palliative care; IC: intensive care; HLHS: hypoplastic left heart syndrome; IUGR: intra-uterine growth restriction; VSD: Ventricular Septal Defect; DORV: Double Outlet Right Ventricle; PA: Pulmonary Atresia; TOF: Tetralogy of Fallot; ARPKD: Autosomal Recessive Polycystic Kidney Disease; ASD: Atrial Septal Defect; LUTO: Lower Urinary Tract Obstruction; PSVT: Paroxysmal Supraventricular Tachycardia; TGV: Transposition of the Great Vessels.

**Table 3:** Study population: Postnatal referral.

N° Patients	Sex	Prenatal Diagnosis	Outcome	Prenatal Plan of Care	Year of Birth
1	F	Glioblastoma	9 months	IC to CC	2014
2	M	Hypoxic–ischemic encephalopathy	17 days	IC to CC	2016
3	M	Mitochondrial disorder	808 days	IC to CC	2017
4	M	Extreme prematurity (23 weeks GA)	24 h	IC to CC	2017
5	M	Mitochondrial disorder	3 months	IC to CC	2018
6	M	Extreme prematurity (23 weeks GA), severe RDS, NEC	44 days	IC to CC	2018
7	F	Extreme prematurity (23 weeks GA)	27 h	IC to CC	2018
8	F	Prematurity (33 weeks GA), hypoxic–ischemic injury	still alive	IC to CC	2019
9	M	Mitochondrial disorder	3 months	IC to CC	2019
10	M	Prematurity, cerebral hemorrhage	2 days	IC to CC	2020
11	M	ALG3-congenital disorder of glycosylation	24 days	IC to CC	2021
12	F	Mitochondrial disorder (Deficit coenzyme Q)	3 months	IC to CC	2022
13	M	Zellweger Syndrome	6 months	IC to CC	2023
14	M	Hypoxic–ischemic encephalopathy	4 days	IC to CC	2024
15	M	Extreme prematurity (23 weeks GA)	50 h	IC to CC	2024
16	M	Hypoxic–ischemic encephalopathy	7 days	IC to CC	2024
17	F	Zellweger Syndrome, prematurity, NEC	35 days	IC to CC	2025
18	M	Extreme prematurity (24 weeks GA)	23 days	IC to CC	2025
19	F	Prematurity (29 weeks), NEC totalis	18 days	IC to CC	2025

CC: comfort/palliative care; IC: intensive care; RDS: Respiratory Distress Syndrome; NEC: Necrotizing Enterocolitis; GA: Gestational Age.

## Data Availability

The data are available from the corresponding author upon reasonable request and subject to institutional approval.
